# A Naturalistic, European Multi-Center Clinical Study of Electrodermal Reactivity and Suicide Risk Among Patients With Depression

**DOI:** 10.3389/fpsyt.2021.765128

**Published:** 2022-01-05

**Authors:** Vladimir Carli, Gergo Hadlaczky, Nuhamin Gebrewold Petros, Miriam Iosue, Patrizia Zeppegno, Carla Gramaglia, Mario Amore, Enrique Baca-Garcia, Anil Batra, Doina Cosman, Philippe Courtet, Guido Di Sciascio, Joakim Ekstrand, Hanga Galfalvy, Ricardo Gusmão, Catarina Jesus, Maria João Heitor, Miguel Constante, Pouya Movahed Rad, Pilar A. Saiz, Marcin Wojnar, Marco Sarchiapone

**Affiliations:** ^1^National Centre for Suicide Research and Prevention of Mental Ill-Health (NASP), Karolinska Institute, Stockholm, Sweden; ^2^Department of Translational Medicine, Azienda Ospedaliero Universitaria Maggiore della Carità, University of Piemonte Orientale, Novara, Italy; ^3^Clinica Psichiatrica, DINOGMI, University of Genoa, Genoa, Italy; ^4^Department of Psychiatry, Fundacion Jimenez Diaz University Hospital, Autonomous University of Madrid, Madrid, Spain; ^5^Department of Psychiatry and Psychotherapy, University Hospital of Tuebingen, Tuebingen, Germany; ^6^Clinical Psychology and Mental Health Department, Iuliu Hatieganu University of Medicine and Pharmacy, Cluj-Napoca, Romania; ^7^Department of Emergency Psychiatry and Acute Care, University Hospital of Montpellier, Montpellier, France; ^8^Department of Mental Health, ASL BARI, Bari, Italy; ^9^Department of Psychiatry, Institute of Clinical Sciences, Lund University, Lund, Sweden; ^10^Department of Psychiatry, Columbia University Medical Center, New York, NY, United States; ^11^Department of Psychiatry, Hospital Egas Moniz, Centro Hospitalar de Lisboa Ocidental (CHLO), Lisbon, Portugal; ^12^Instituto de Saúde Pública, Universidade Do Porto (ISPUP), Porto, Portugal; ^13^Psychiatry Service, Hospital Beatriz Ângelo (HBA), Loures, Portugal; ^14^Department of Psychiatry, Biomedical Research Networking Centre in Mental Health (CIBERSAM), Instituto de Investigación Sanitaria del Principado de Asturias (ISPA), Mental Health Services of Principado de Asturias (SESPA), University of Oviedo, Oviedo, Spain; ^15^Department of Psychiatry, Medical University of Warsaw, Warsaw, Poland; ^16^Department of Medicine and Health Sciences, University of Molise, Campobasso, Italy

**Keywords:** electrodermal activity (EDA), depression, suicide, suicidal behavior, suicide attempt, suicide risk assessment

## Abstract

**Background:** Electrodermal hyporeactivity has been proposed as a marker of suicidal risk. The EUDOR-A study investigated the prevalence of electrodermal hyporeactivity among patients with depression and its association with attempted and completed suicide.

**Methods:** Between August 2014 and March 2016, 1,573 in- and outpatients with a primary diagnosis of depression (active or remission phase) were recruited at 15 European psychiatric centers. Each patient was followed-up for 1 year. Electrodermal activity was assessed at baseline with the ElectroDermal Orienting Reactivity Test. Data on the sociodemographic characteristics, clinical diagnoses, and treatment of the subjects were also collected. The severity of the depressive symptoms was assessed through the Montgomery–Asberg Depression Rating Scale. Information regarding number, time, and method of suicide attempts was gathered at baseline and at the end of the 1-year follow-up. The same data were collected in case of completed suicide.

**Results:** Hyporeactive patients were shown to be significantly more at risk of suicide attempt compared to reactive patients, both at baseline and follow-up. A sensitivity of 29.86% and a positive predictive value (PPV) of 46.77% were found for attempted suicide at baseline, while a sensitivity of 35.36% and a PPV of 8.92% were found for attempted suicide at follow-up. The sensitivity and PPV for completed suicide were 25.00 and 0.61%, respectively. However, when controlled for suicide attempt at baseline, the association between hyporeactivity and follow-up suicide attempt was no longer significant. The low number of completed suicides did not allow any analysis.

## Introduction

Suicidal behavior is one of the most common and serious psychiatric emergencies (1), and suicide risk assessment is one of the main challenges for mental health professionals (2). However, valid and reliable objective methods to support suicide risk assessment are still lacking (3).

Electrodermal activity (EDA) refers to electrical events in the skin caused by the activity of eccrine sweat glands in palmar skin (4). Variations in the sweating of the skin are regulated by environmental temperature (thermoregulatory sweating) and by central nervous activity related to affective and cognitive states (palmar or emotional sweating) (5, 6).

EDA is characterized by a tonic and phasic component. The tonic component represents the basic level of conductance, while the phasic component is related to the faster changing elements of the signal that can be associated with a stimulus or be “spontaneous” or “non-specific” (7, 8).

The amplitude of the electrodermal response increases linearly with perceived arousal when subjects are exposed to emotional stimuli (9–12). Electrodermal reactivity refers to responses to a repeated identical neutral tone stimulus. The repeated presentation of identical and non-significant stimuli elicits progressively smaller reactions, a learning process known as habituation (8, 13). Nevertheless, some subjects show unusual rapid habituation, and for this reason, they are defined as hyporeactive.

Many studies have focused on electrodermal hyporeactivity as a possible biomarker for depression (14–22), and it has been suggested that differences in electrodermal reactivity may be specific to suicidality rather than depression (23–27). In particular, both violent suicide attempters and suicide completers were shown to be fast habituators (24, 28, 29).

Further studies indicated a correlation between electrodermal reactivity and the type and level of suicide risk. A meta-analysis based on 297 depressed patients and 59 healthy subjects concluded that electrodermal hyporeactivity is strongly associated with a high suicide risk. The authors reported a sensitivity of 96.6% and a negative predictive value (NPV) of 92.9% for suicide and a sensitivity of 83.3% and a NPV of 92.7% for suicide and/or violent attempt, thus indicating that a test of electrodermal hyporeactivity may have a high discriminative validity for vulnerability to suicide (30). Another study on 783 depressed patients confirmed that electrodermal hyporeactivity may be a marker of suicidal tendency in depression, independently of gender, age, depression severity, and trait anxiety (31).

Overall, the most recent review available about the EDA literature reported that, despite the features and quality of the studies included (quite outdated and employing a wide variety of designs), electrodermal hyporeactivity seemed to be a reliable feature of depression and a potentially useful marker of suicidal risk (32).

The EUDOR-A study was aimed at assessing the effectiveness and the usefulness of the ElectroDermal Orienting Reactivity (EDOR) Test as a support in the suicide risk assessment of depressed patients and to assess the predictive value of electrodermal hyporeactivity, measured through the EDOR Test, for suicide and suicide attempt in adult patients with a primary diagnosis of depression (33). With this purpose, data were collected about (1) the prevalence of electrodermal hyporeactivity among patients with depression and (2) the incidence of actions of intentional self-harm, i.e., completed suicide after, and suicide attempt before and after test during the 1-year follow-up period. The aim of the current analysis is to assess the predictive value of electrodermal hyporeactivity, measured through the EDOR Test, for suicide and suicide attempt in adult patients with a primary diagnosis of depression. We hypothesized that the EDOR Test would identify electrodermal hyporeactive depressed patients with a higher suicidal proneness as indicated by intentional self-harm behaviors.

## Materials and Methods

### Study Design and Patient Characteristics

The design of the EUDOR-A naturalistic, observational study has been thoroughly described elsewhere, including sample size calculation (33). The EUDOR-A study was funded by Emotra AB, Sweden. It was registered in the German Clinical Trials Registry (DRKS00010082) and received ethical approval at each study site.

**Figure 1:**
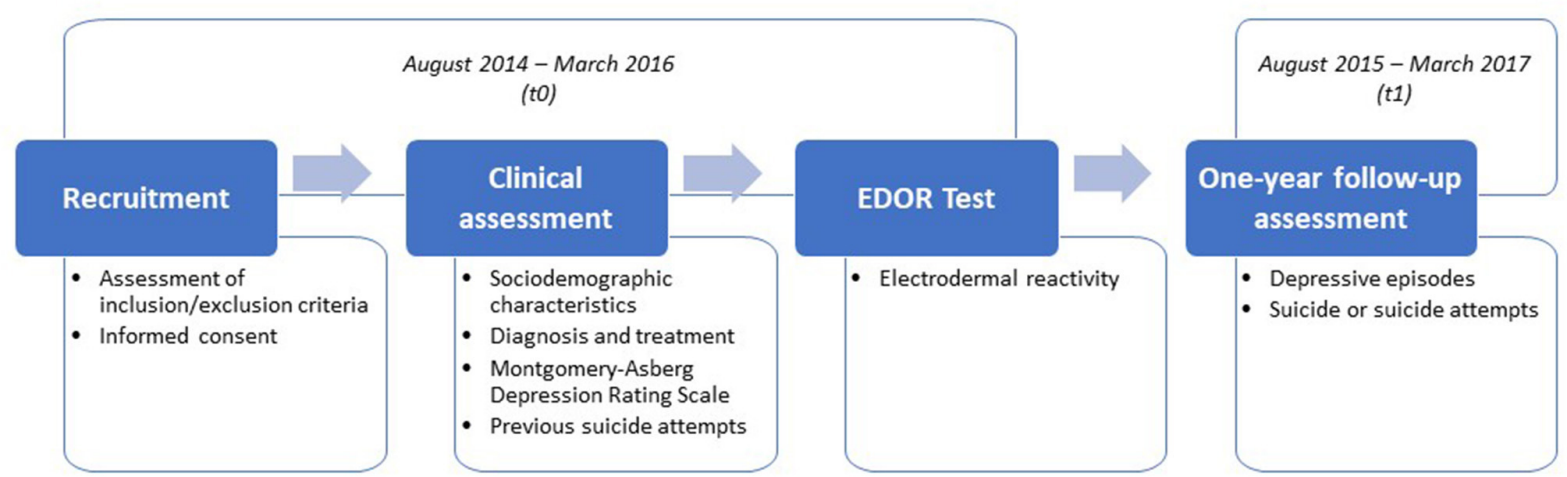
EUDOR-A study design.

Patients were recruited at 15 psychiatric clinics in nine different European countries. Recruitment took place from August 2014 to March 2016 (t0) (Figure 1). All in- and out-patients with the following primary diagnoses, also in remission, were invited to participate. The included primary psychiatric diagnoses, according to the International Classification of Diseases, Tenth Revision (ICD-10) (34), were bipolar disorder (F31.3–F31.9), depressive episode (F32.0–F32.9), recurrent depressive disorder (F33.0–F33.9), persistent mood disorder (F34.0, F34.1, F34.8, and F34.9), other mood disorders (F38.0, F38.1, and F38.8), and unspecified mood disorder (F39). Patients were included if they were 18 years old or older and gave written informed consent to participate in the study. Patients were excluded if they were unable to understand the instructions for the EDOR Test, had severe hearing problems, or had dementia, history of drug, alcohol, or any type of abuse as listed in the protocol paper (33). Patients were also excluded if they were not willing to participate at any time during the study.

A comprehensive clinical assessment was performed to collect information concerning the sociodemographic characteristics of the patients, diagnosis and treatment, and previous suicide attempts. Immediately after the assessment, the EDOR Test was performed. Each enrolled patient was then followed-up for 1 year (from August 2015 to March 2017) (t1).The final recruited sample included 1,573 in- and outpatients with a primary diagnosis of depression (active or remission phase).

**Figure 2:**
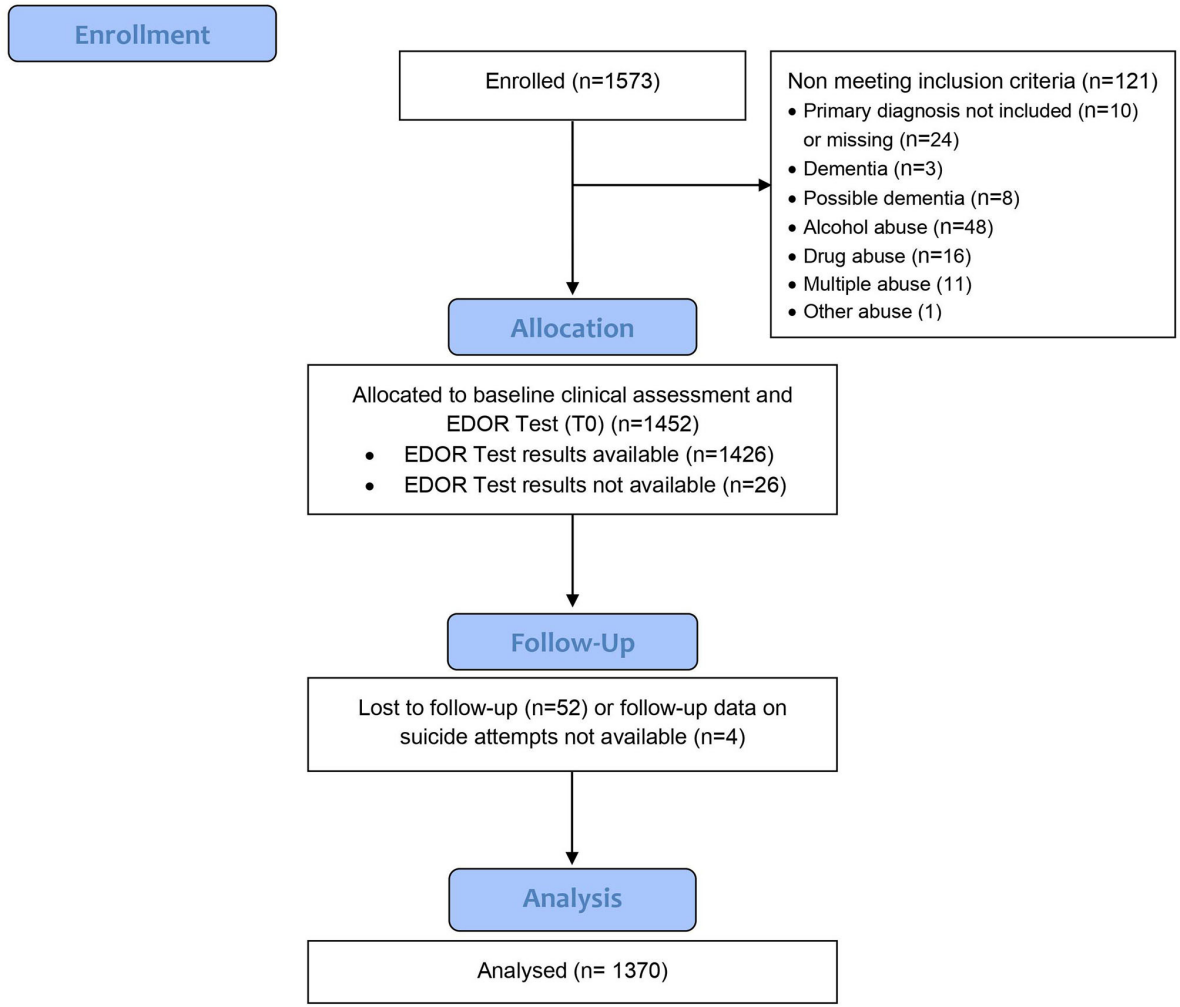
CONSORT flow diagram–modified.

The CONSORT flow diagram, modified for a non-randomized trial, is shown in Figure 2.

### Assessment and Measures

#### Reactivity

Electrodermal reactivity was assessed at baseline, t0, with the EDOR Test. The EDOR Test (23, 26) was developed by Thorell at the beginning of the 1980s. Since then, the test device has been commercialized by the Swedish company Emotra AB and modified to facilitate the use of the device by any member of the clinical staff at the ward after a short training session.

Since previous research has reported that hyporeactivity is independent of time of day (23), the EDOR Test can be performed at any time of a day in a quiet stimuli-free room. A test session takes about 25 min, including a 15-min-long habituation test. The EDOR Test uses a series of neutral audio tones as stimuli and then measures the presence or absence of specific orienting reactions in the sweating system recorded as electrodermal responses. Electrodermally hyporeactive individuals are those who show an unusually rapid habituation. The EDOR Test has been described in detail elsewhere (33).

Once the test was finished, pseudoanonymized and encrypted data were sent over the Internet for remote analyses at Emotra AB. The experts who analyzed the test data were blinded to the clinical assessment of the patients. After the analyses, a test report was sent back to the predefined e-mail addresses of the authorized staff at the remitting center.

The test report contained information on the quality of the assessment and four categories of reactivity: hyporeactivity, on the verge of hyporeactivity, reactivity, and hyperreactivity. Based on previous literature (31) and since the “hyperreactive” and “on the verge of hyporeactive” categories included very few subjects, the sample was dichotomized into two groups. Hyporeactive and on the verge of hyporeactive were included in one group, collectively called hyporeactive. Hyperreactive and reactive were included in the second group, collectively called reactive. It should be noted that this dichotomization did not yield significant changes on the OR, sensitivity, or specificity.

#### Suicide Attempts and Completed Suicide

Suicide attempt was defined as an act of intentional self-harm with the intent to die (33, 35).

Information regarding previous suicide attempts, including number, time, and method (assigning appropriate ICD-10 codes) of suicide attempts, was collected using a semi-structured interview administered at baseline (t0), before the EDOR Test. Suicide attempt before the EDOR Test was recorded as a yes or no variable.

The same variables were collected at the 1-year follow-up (t1) at each center using a similar semi-structured interview, administered in person or by phone. Completed suicide during follow-up was also recorded as a yes or no variable, the method used was recorded, and appropriate ICD-10 codes were assigned.

With regards to suicide attempts and completed suicides, the use of a violent method was recorded as a yes or no variable.

#### Other Variables

Data on socio-demographics, clinical diagnoses (primary and secondary psychiatric and somatic diagnoses), and treatment (pharmacological and psychological) of the participants were gathered at baseline.

Assessment of depression was performed with the clinician-rated Montgomery–Asberg Depression Rating Scale (MADRS) at t0 (36, 37). The scale is composed of 10 items rated on a seven-point Likert scale (0–6), with higher scores reflecting more severe symptoms of depression. It was developed to measure the severity of depressive episodes in patients with mood disorders and quantify apparent sadness, reported sadness, inner tension, reduced sleep, reduced appetite, concentration difficulties, lassitude, inability to feel, pessimistic thoughts, and suicidal thoughts. The MADRS score was used as an indicator of depression severity.

### Statistical Analyses

The utilized quantitative measures (age and MADRS) were graphed to look for outliers or inconsistent values, and none was found. The association between hyporeactive status and baseline sociodemographic and clinical measures was tested using chi-square tests of independence, Fisher's exact tests, *t*-tests, or Mann–Whitney *U*-tests as appropriate. The association between hyporeactivity and the method used for the suicide attempt was tested using a chi-square test.

The sensitivity, specificity, and positive and negative predictive values of the EDOR Test were calculated for suicide attempts before the test, suicide attempts during follow-up, and completed suicide during follow-up. These analyses were also performed separately for patients with a diagnosis of unipolar depression (F32 and F33) and bipolar depression (F31). Receiver operating characteristic (ROC) curves were conducted to look at the area under the ROC curve (AUC) for each outcome.

Furthermore, a logistic regression was conducted to estimate the increased risk of suicide attempt for hyporeactive patients. Odds ratios (OR) of suicide attempt were estimated using the log-binomial model in reactive and hyporeactive patients, adjusting for sex, age, type of care, use of antidepressant, and primary psychiatric diagnosis. To further investigate and compare the information that the EDOR Test provides, the OR of suicide attempt at follow-up was estimated, adjusting for history of suicide attempt. All analyses were conducted using SPSS v25 and at an alpha level of 0.05.

#### Ethical Considerations

The study protocol received ethical approval from the local ethics committee in each participating center where the research project was performed. The participants received information from their clinicians about the study and that the EDOR Test was being studied as a new tool for helping clinicians in assessing the risk of suicide in depressed patients. They were also informed that the results of the test would be interpreted by an independent expert and then communicated to their physicians. A signed informed consent form was obtained from each patient. To ensure confidentiality, data was pseudoanonymized. There was unanimous agreement among all researchers and the ethical committees that it would be unethical, based on the results from previous research (30, 38), to recruit a control group of patients whose clinicians were blinded to the test results. Therefore, each center was free to use the results of the EDOR Test in their clinical suicide risk assessments and treatment plan for participating patients.

## Results

### Characteristics of the Studied Sample

Overall, data from 87.09% (*N* = 1,370) of the total sample were analyzed. One hundred fifty one (9.60%) patients were excluded due to missing data or because of not meeting the inclusion criteria. Additionally, 52 (3.30%) patients were excluded from the analyses because they were lost to follow-up (Figure 1). However, no significant differences were found among the patients lost to follow-up and the rest of those included in the analyses with regards to age, gender, inpatient status, psychiatric and somatic diagnoses, MADRS scores, reactivity, and suicidal attempts before the EDOR Test. Described in Table 1 are the characteristics of the study population.

**Table 1:** Sociodemographic and clinical characteristics of the study population at baseline.

	**Total**	**Suicide attempt baseline**	**Suicide attempt during follow-up**	**Completed suicide**
Total (%)	1,370	509 (37.15)	82 (5.98)	8 (0.58)
Male (%)	434 (31.68)	138 (27.11)	30 (36.58)	5 (62.50)
Mean age (SD)	50.06 (14.49)	47.32 (14.26)	44.93 (14.05)	56.10 (18.35)
Patients in inpatient care (%)	672 (49.05)	304 (59.72)	58 (70.73)	6 (75.00)
Electrodermal reactivity (%)				
Hyperreactive	8 (0.58)	4 (0.78)	0	0
Reactive	1,037 (75.69)	353 (69.35)	53 (64.63)	6 (75)
On the verge of being hyporeactive	66 (4.82)	23 (4.52)	6 (7.32)	0
Hyporeactive	259 (18.90)	129 (25.34)	23 (28.05)	2 (25)
Primary psychiatric diagnosis (%)				
Bipolar disorder (F31)	294 (21.46)	132 (25.93)	27 (32.93)	1 (12.50)
Major depressive disorder, single episode (F32)	447 (32.63)	150 (29.47)	24 (29.27)	5 (62.50)
Major depressive disorder, recurrent episodes (F33)	528 (38.54)	183 (35.95)	25 (30.49)	1 (12.50)
Persistent mood disorder (F34)	97 (7.08)	41 (8.05)	6 (7.32)	1 (12.50)
Unspecified mood disorder (F39)	4 (0.29)	3 (0.59)	0	0
Secondary psychiatric diagnosis (%)	316 (23.06)	149 (29.27)	26 (31.71)	2 (25.00)
Somatic disorder (%)	493 (35.98)	173 (33.99)	19 (23.17)	2 (25.00)
Mean MADRS (SD)	22.09 (10.54)	24.16 (10.12)	26.13 (10.71)	23.75 (9.85)
Antidepressant (%)	1,138 (83,06)	423 (83.10)	66 (80.49)	8 (100.00)
Psychotherapy (%)	220 (16.06)	65 (12.77)	13 (15.85)	0

The cohort had a higher proportion of female patients (*n* = 935; 68.25%) than male (*n* = 434; 31.68%), and one participant had missing gender data. Furthermore, 37.15% of the study population had attempted suicide at baseline (before the EDOR Test), where 32.02% (*n* = 163) used a violent method (not shown). Five patients had missing data regarding suicide attempt method at baseline. A total of 82 patients (5.98%) attempted suicide during follow-up, and 20 patients (24.10%) used a violent method (not shown). Four patients had missing data regarding suicide attempt method during follow-up. Eight patients (0.58%) died by suicide during the follow-up period. Of the total population, 75.69% had reactive electrodermal activity, with only 0.58% testing hyperreactive. Majority of the patients (38.54%) had a diagnosis of F33 (major depressive disorder, recurrent) followed by F32 (major depressive disorder, single episode) where 32.63% were diagnosed. A total of 21.46% had a diagnosis of F31 (bipolar disorder), while 23.06 and 35.98% also had a secondary psychiatric and somatic diagnosis, respectively. Moreover, at baseline, 83.06% were currently treated with antidepressants, whereas 16.06% were treated with psychotherapy. Out of these participants, 15.18% (*n* = 179) received both antidepressants and psychotherapy.

There were significant differences with regards to age (*p* < 0.001), type of care (*p* < 0.001), primary psychiatric diagnosis (*p* < 0.01), and MADRS total scores (*p* < 0.001) in patients who attempted suicide at baseline compared to the study population.

Table 2 illustrates the different characteristics of patients who attempted suicide before and after EDOR Test with respect to electrodermal activity.

**Table 2:** Characteristics of the study population by reactivity.

**Total population**	**Reactive** ***n* = 1,045**	**Hyporeactive** ***n* = 325**
Male (%)	331 (31.67)	103 (31.69)
Mean age (SD)	50.00 (14.43)	50.27 (14.73)
Patients in inpatient care at t0 (%)	503 (48.13)	169 (52.00)
Primary psychiatric diagnosis (%)		
Bipolar disorder (F31)	204 (19.52)	90 (27.69)
Major depressive disorder, single episode (F32)	349 (33.40)	98 (30.15)
Major depressive disorder, recurrent episodes (F33)	417 (39.90)	111 (34.15)
Persistent mood disorder (F34)	72 (6.89)	25 (7.69)
Unspecified mood disorder (F39)	3 (0.29)	1 (0.31)
Mean MADRS at t0 (SD)	21.73 (10.50)	23.25 (10.60)
**Population with suicide attempts at baseline**	**Reactive:** ***n*** **= 357**	**Hyporeactive:** ***n*** **= 152**
Male (%)	97 (27.17)	41 (26.97)
Mean age (SD)	47.71 (14.44)	46.38 (13.81)
Patients in inpatient care at t0 (%)	214 (59.94)	90 (59.21)
Patients with recurrent depressive disorder (%)	140 (39.21)	43 (28.29)
Mean MADRS at t0 (SD)	23.68 (10.29)	25.28 (9.91)
**Population with suicide attempts at follow-up**	**Reactive:** ***n*** **= 53**	**Hyporeactive:** ***n*** **= 29**
Male (%)	19 (35.85)	11 (37.93)
Mean age (SD)	45.74 (13.74)	43.44 (14.73)
Patients in inpatient care at t0 (%)	39 (73.58)	19 (65.52)
Patients with recurrent depressive disorder (%)	20 (37.73)	5 (17.24)
Mean MADRS at t0 (SD)	26.23 (11.46)	25.97 (9.36)

### Prevalence of Hyporeactivity

Of the total population, 76.28% (*N* = 1,045) tested reactive (Table 2). A similar proportion of male patients was classified as reactive and hyporeactive, with a percentage of 31.67 and 31.69%, respectively. The mean age in these subgroups (50 and 50.27 in reactive and hyporeactive, respectively) was also similar to the total study population. In the total sample, a similar distribution with respect to primary psychiatry diagnosis was observed among reactive and hyporeactive patients where 39.90 and 34.15% were diagnosed with recurrent depressive disorder, respectively (Table 2).

There was no statistical difference in the demographic (age and gender) or clinical (inpatient care, primary diagnosis, and MADRS scores) variables between patients who were reactive and hyporeactive in the group of patients that had already attempted suicide at baseline and the group that attempted suicide during follow-up (*p* > 0.05, not shown).

### Hyporeactivity and Suicide Attempts

There was a statistically significant difference (*p* < 0.001), tested with chi-square, between the number of patients classified as hyporeactive and reactive with regards to suicide attempt before the EDOR Test. The EDOR Test had high specificity (79.91%) but lower sensitivity (29.86%) coupled with a lower positive predictive value (PPV) value (46.77) as well (Table 3). The EDOR Test showed no discriminatory ability for previous suicide attempts (AUC = 0.549).

**Table 3:** Sensitivity, specificity, and AUC of ElectroDermal Orienting Reactivity (EDOR) test and suicide attempt at baseline.

		**Attempted suicide at baseline^a^**				
		**Yes**	**No**	**Total**		**Value (95% CI)**
EDOR Test	Hyporeactive	TP = 152	FP = 173	325	Sensitivity (%)	29.86 (25.92–34.05)
	Reactive	FN = 357	TN = 688	1,045	Specificity (%)	79.91 (77.07–82.54)
	Total	509	861	1,370	PPV (%)	46.77 (42.12–51.47)
					NPV (%)	65.84 (64.34–67.30)
					AUC	0.549 (0.517–0.591)

*TP, true positive; FP, false positive; FN, false negative; TN, true negative; AUC, area under the curve.*

a *p < 0.001*.

Similarly, there was a statistically significant difference (*p* < 0.05) between the number of patients classified as hyporeactive and reactive with regards to suicide attempts at follow-up, and sensitivity was higher for risk of suicide attempt at follow-up than at baseline. However, the PPV value was much lower for suicide attempts during follow-up than for suicide attempts at baseline, and the discriminatory ability of the test did not improve (Table 4).

**Table 4:** Sensitivity, specificity, and AUC of ElectroDermal Orienting Reactivity (EDOR) test and suicide attempt during follow-up.

		**Attempted suicide during follow-up^a^**				
		**Yes**	**No**	**Total**		**Value (95% CI)**
EDOR Test	Hyporeactive	TP = 29	FP = 296	325	Sensitivity (%)	35.36 (25.12–46.70)
	Reactive	FN = 53	TN = 992	1,045	Specificity (%)	77.02 (74.62–79.29)
	Total	82	1,288	1,370	PPV (%)	8.92 (6.71–11.78)
					NPV (%)	94.93 (94.08–95.66)
					AUC	0.562 (0.495–0.629)

*TP, true positive; FP, false positive; FN, false negative; TN, true negative; AUC, area under the curve.*

a *p = 0.011*.

Several patients who attempted suicide before the EDOR Test also attempted suicide during follow-up. Therefore, an analysis was conducted excluding patients with a previous suicide attempt (Table 5).

**Table 5:** Sensitivity, specificity, and AUC of ElectroDermal Orienting Reactivity (EDOR) Test and suicide attempt during follow-up, excluding patients with a previous suicide attempt.

		**Attempted suicide during follow-up^a^**				
		**Yes**	**No**	**Total**		**Value (95% CI)**
EDOR Test	Hyporeactive	TP = 6	FP = 167	173	Sensitivity (%)	40.00 (16.34–67.71)
	Reactive	FN = 9	TN = 679	688	Specificity (%)	80.26 (77.42–82.89)
	Total	15	846	861	PPV (%)	3.47 (1.87–6.35)
					NPV (%)	98.69 (98.03–99.13)
					AUC	0.601 (0.446–0.757)

*TP, true positive; FP, false positive; FN, false negative; TN, true negative; AUC, area under the curve.*

a *Non-significant*.

Hyporeactivity was not associated with the use of a violent suicide attempt method nor at baseline or during the follow-up.

There were eight completed suicides during the follow-up period. There were no statistically significant differences in the number of completed suicide between the hyporeactive and the reactive groups (Table 6).

**Table 6:** Sensitivity, specificity, and AUC of ElectroDermal Orienting Reactivity (EDOR) test and suicide.

		**Suicide^a^**				
		**Yes**	**No**	**Total**		**Value (95% CI)**
EDOR Test	Hyporeactive	TP = 2	FP = 323	325	Sensitivity (%)	25.00 (3.19–65.09)
	Reactive	FN = 6	TN = 1,039	1,045	Specificity (%)	76.28 (73.93–78.52)
	Total	8	1,362	1,370	PPV (%)	0.61 (0.19–2.02)
					NPV (%)	99.42 (99.14–99.61)
					AUC	0.506 (0.304–0.708)

*TP, true positive; FP, false positive; FN, false negative; TN, true negative; AUC, area under the curve.*

a *Non-significant*.

All analyses of sensitivity and specificity were also performed separately for patients with a diagnosis of unipolar depression or bipolar disorder (Supplementary Tables 1–4). The association between hyporeactivity and suicide attempt at baseline was significant for patients with unipolar but not bipolar depression. The association with suicide attempt at follow-up was significant for bipolar but not for unipolar depression.

The relative risk of suicide attempt in relation to electrodermal reactivity was calculated by controlling for demographic and clinical covariates. Overall, patients who were classified as hyporeactive had a statistically significant increased risk (OR 1.69, 95% CI: 1.31–2.18) of suicide attempts at baseline (Table 7). Adjusting for age, sex, type of care, use of antidepressant, and primary psychiatric diagnosis did not yield a different odds ratio.

**Table 7:** Odds ratio (OR) and 95% confidence intervals (CI) of the association between attempted suicide at baseline and hyporeactivity.

	***N* ^a^**	***n* ^b^**	**Crude estimate**	**Adjusted estimate**
			**OR (95% CI)**	**OR^c^ (95% CI)**
Hyporeactive	325	152	1.69 (1.31–2.18)	1.67 (1.28–2.16)

a *Number of hyporeactive patients.*

b *Number of hyporeactive patients with attempted suicide at baseline.*

c *Adjusted for age, sex, type of care, use of antidepressant and primary psychiatric diagnosis*.

Similarly, there was also a statistically significant increased risk (OR 1.83, 95% CI: 1.14–2.94) of suicide attempt during follow-up in hyporeactive patients compared to reactive patients (Table 8). The risk estimate did not change when it was adjusted for several covariates.

**Table 8:** Odds ratio (OR) and 95% confidence intervals (CI) of the association between attempted suicide at follow-up and hyporeactivity.

	***N* ^a^**	***n* ^b^**	**Crude estimate**	**Adjusted estimate**
			**OR (95% CI)**	**OR^c^ (95% CI)**
Hyporeactive	325	29	1.83 (1.14–2.94)	1.72 (1.06–2.79)

a *Number of hyporeactive patients.*

b *Number of hyporeactive patients with attempted suicide at follow-up.*

c *Adjusted for age, sex, type of care, use of antidepressant, and primary psychiatric diagnosis*.

An OR was estimated for suicide attempts at follow-up using both hyporeactivity and history of suicide attempts as independent variables (Table 9). The OR for hyporeactivity was not significant (OR 1.47, 95% CI: 0.90–2.40) when history of suicide attempts was added to the model, and this did not change when adjusting for demographic and clinical covariates. However, the OR for suicide attempt at baseline was significant (OR 8.24, 95% CI: 4.64–14.62) when reactivity was added to the model, and the estimate remained significant when demographic and clinical covariates were included in the logistic model.

**Table 9:** Odds ratio (OR) and 95% confidence intervals (CI) of the association between attempted suicide at follow-up using hyporeactivity and attempted suicide at baseline as independent variables.

	***N***	***n***	**Crude estimates**	**Adjusted estimates**
			**OR^a^ (95% CI)**	**OR^b^ (95% CI)**
Hyporeactive	325^c^	29^d^	1.47 (0.90–2.40)	1.42 (0.86–2.32)
Attempted suicide at baseline	509^e^	67^f^	8.24 (4.64–14.62)	7.28 (4.06–13.05)

a *Adjusted for attempted suicide at baseline for reactivity and adjusted for reactivity for attempted suicide at baseline.*

b *Adjusted for attempted suicide at baseline (for reactivity), reactivity (for attempted suicide at baseline), age, sex, type of care, use of antidepressant, and primary psychiatric diagnosis.*

c *Number of hyporeactive patients.*

d *Number of hyporeactive patients with attempted suicide at follow-up.*

e *Number of patients with attempted suicide at baseline.*

f *Number of patients with attempted suicide at baseline and at follow-up*.

## Discussion

This study found a statistically significant increased risk of suicide attempt at baseline and suicide attempt at follow-up in hyporeactive patients compared to reactive patients. The risk of suicide attempts after 1 year among hyporeactive patients was almost twofold the risk of suicide attempts at follow-up among reactive patients. This risk also did not significantly change when we adjusted for demographic and clinical covariates. Hyporeactivity was no longer significantly associated with a higher risk of suicide attempt at follow-up when suicide attempt at baseline was added to the regression model. Suicide attempt at baseline instead turned out to be a significant risk factor for future suicide attempts in comparison to hyporeactivity, where patients with a history of attempted suicide had almost eight times higher risk for a future suicide attempt compared to patients with no history of suicide attempt. The odds ratio for completed suicide was not computed due to the low number of suicides during the 1-year follow-up. Even if the increased suicide attempt risk associated with hyporeactivity is significant from a statistical point of view, its small effect size does not allow one to make a meaningful clinical decision about a specific patient. Moreover, a previous suicide attempt appears to be a much stronger factor than hyporeactivity. Overall, the results of this study show that hyporeactivity, as a standalone measure, has a limited clinical significance for the determination of suicide risk.

The sensitivity, NPV, and PPV of the EDOR Test were lower than in previous studies (30, 31). In a meta-analysis including 279 depressed patients and 59 healthy subjects, the sensitivity of electrodermal hyporeactivity for suicide was 96.6%, and the specificity was 92.9%, while the sensitivity and specificity for suicide and violent suicide attempts was 92.7 and 83.3%, respectively (30). Similarly, data from 892 depressed patients collected between 1985 and 2002 in Germany yielded a sensitivity of 83.3% and a specificity of 97.6% for suicide. In the current study, a sensitivity of 29.86% and a PPV of 46.77% were found for attempted suicide at baseline, while a sensitivity of 35.36% and a PPV of 8.92% were found for attempted suicide at follow-up. The sensitivity and PPV of reactivity for completed suicide was 25.00 and 0.61%, respectively. Even if specificity was around 80% for all the outcome measures, this result should be considered cautiously since it may be due to the combination of a relatively rare outcome (i.e., suicidal behavior) with the low prevalence of hyporeactivity in the sample (39).

However, the results should be discussed in the light of some relevant strengths, limitations, and considerations about the study. The study has several strengths. While previous investigations were conducted using smaller samples recruited in a single psychiatric center (30, 31), the EUDOR-A study recruited patients from 15 psychiatric centers in nine countries. The subjects were already out- or inpatients of the clinics and did not receive any incentive to take part in the study. Moreover, the sociodemographic characteristics of the sample, as well as the reported prevalence of suicidal behavior, are in line with literature findings. The higher proportion of female patients compared to male patients (68.25 vs. 31.68%) in the study sample is very similar to the female–male ratio of 2:1 described in epidemiological studies and meta-analyses on depressed patients (40, 41). The overrepresentation of female patients among suicide attempters found in this sample has been previously described in other research as the “gender paradox” (42, 43) since it is usually coupled with a preponderance of male patients in completed suicides. Furthermore, the proportion of female patients (68.25%) in our sample is similar to that reported in other studies investigating the relationships between EDA and suicidal behavior (24, 29, 44), and hyporeactivity seems to be independent from sex (29, 31).

In the current study, 37.15% (*n* = 509) of the sample reported a lifetime history of suicide attempt at baseline. Since the study sample mostly includes patients with a diagnosis of major depression, this result can be considered very similar to the one reported in epidemiological studies on depressed patients. Comparing the results of two large research projects on bipolar and depressed patients in Finland, Holma et al. (45) found that, before baseline, 51% of patients with bipolar disorder and 33% of patients with major depression disorder had attempted suicide. In his review on suicidal behavior in mood disorders, Isometsä (46) reported the lifetime prevalence of suicide attempt to be 30–40% in major depression and about 50% in bipolar out- and inpatients. More recently, Dong et al. (47, 48) conducted two systematic reviews and meta-analyses of observational studies to estimate the prevalence of suicide attempts among patients with major depression and bipolar disorders. They calculated a pooled lifetime prevalence of suicide attempts of 31% (95% CI: 27–34%; *I*^2^ = 97.2%) and 33.9% (95% CI: 31.3–36.6%; *I*^2^ = 96.4%), respectively. During the 1-year follow-up, around 6% (*n* = 82) of the EUDOR-A sample attempted suicide. In the study by Holma et al. (44), 19.9% of bipolar patients and 9.5% of major depressed patients attempted suicide during the 18-month follow-up. In the meta-analyses conducted by Dong et al. (47, 48), the 1-year prevalence of suicide attempts was 15.0% for bipolar disorder and 8% for major depression.

Nevertheless, the small sample size and short follow-up limit the findings of this study. More specifically, the incidence of completed suicide was low (eight suicides during the 1-year follow-up), and a longer follow-up might have yielded more completed suicides, thus improving the power of the analysis. Indeed in the study by Thorell et al. (31) reporting higher sensitivity and specificity, electrodermal and suicide data collected over 17 years were analyzed. The prevalence of hyporeactivity in the overall sample was also much higher than that found in the EUDOR-A sample (68.1 vs. 23.72%). It should also be noted that the test result from the EDOR Test gives a continuous reactivity value, and the four categories (hyporeactive, on the verge of hyporeactive, reactive, and hyperreactive) were made based on cutoff values. However, grouping these categories differently to form two comparative groups did not yield significant changes on the ORs, sensitivity, or specificity.

The major limitation of the EUDOR-A study is the non-blinded design of the study, which was chosen due to ethical reasons. Clinicians were aware of the results of the EDOR Test, which might have led them to increase the intensity of care for patients who were hyporeactive. Therefore, the reduction of attempted suicide at follow-up in hyporeactive patients and thus the increased number of false positives might be due to the above-mentioned increase in the intensity of care—for instance, additional precaution plans and preventive measures might have been put in place due to the expectation from the carers that hyporeactive patients were more prone to suicidal behavior, diminishing the suicidal behavior that would have developed otherwise. Unfortunately, a measure of intensity of care was not included in the protocol of the study, and it is not possible to evaluate if additional suicide preventive measures for hyporeactive patients increased the number of false positives and subsequently decreased the measured sensitivity and specificity of the EDOR Test.

Another limitation lies in the different patterns of association between hyporeactivity and suicidal behaviors shown by unipolar and bipolar depressed patients (see Supplementary Tables 1–4). This is likely due to the small size of the subsamples. Previous studies suggested that there is no difference in tonic or phasic electrodermal activity between unipolar and bipolar depression (49, 50), and therefore unipolar and bipolar depressed patients were considered and studied as a uniform category (28, 30, 31). However, in the current study, bipolar depressed patients were significantly more often classified as hyporeactive than the other diagnostic categories (30.6 vs. 21–25.8%), and Thorell et al. (31) reported an even higher prevalence of hyporeactivity in the subgroup of bipolar patients (80%). Further studies with larger sample sizes are needed to better investigate differences in hyporeactivity among unipolar and bipolar depressed patients and its link to suicidality.

The EUDOR-A study partly confirms previous literature that also reports electrodermal hyporeactivity in depressed patients who attempt suicide compared to non-suicidal depressed patients (23–27, 29, 44). However, the results of the current study stand in contrast with previous research, which support the presence of a correlation between hyporeactivity and the choice of method (violent or non-violent) for attempted or completed suicide (23, 24, 27–31). Literature also suggests that electrodermal hyporeactivity might be a psychophysiological correlate of antisocial behavior, showing a moderate hereditability (51, 52). Future research should investigate if hyporeactivity is a proxy for a brain state that predisposes to aggression and self-harm and evaluate if treatments aimed at preventing suicide may influence electrodermal reactivity.

In line with previous research (23, 29, 31), attempted suicide at baseline was a very significant risk factor in the adjusted model. Other than a history of suicide attempt, no other moderators, such as age, gender, diagnosis, and pharmacological treatment, were found for the association between hyporeactivity and risk of suicide attempt at follow-up.

## Conclusions

The main finding of the EUDOR-A study is that an electrodermal hyporeactive response pattern to neutral stimuli is associated with a history of suicide attempts as well as with an increased risk of such behavior during a 12-month follow-up period after being tested. However, the sensitivity and specificity of the EDOR Test were not sufficient to support its use as a suicide risk assessment tool in clinical practice. The statistically significant association of hyporeactivity with suicide attempts is promising and should be further investigated. Future research should address and overcome the limitations that emerged from the current study, including the inclusion of a control group, with clinicians blinded to the results of the EDOR Test or, if impossible due to ethical reasons, a detailed collection of the data about the preventive interventions applied to hyporeactive and reactive patients, allowing one to measure the intensity of treatment delivered to each patient.

## Data Availability Statement

The raw data supporting the conclusions of this article will be made available by the authors, without undue reservation.

## Ethics Statement

The studies involving human participants were reviewed and approved by Committee to Protect People southern Mediterranean I (Comité de Protection des Personnes Sud Méditerranée I), Marseilles (France), approved on December 5, 2014; Ethics Committee of the Medical Faculty of the University of Rostock (Ethikkommission an der Medizinischen Fakultät der Universität Rostock), Rostock (Germany), approved on August 8, 2014; Ethics Committee of the Medical Faculty of the University of Tuebingen (Ethikkommission an der Medizinischen Fakultät der Eberhard Karls Universität Tübingen), Tuebingen (Germany), approved on September 9, 2014; Regional and Institutional Ethics Committee (Regionális és Intézményi Kutatásetikai Bizottság), Budapest (Hungary), approved on October 31, 2014; Independent Ethics Committee of the University Hospital (Comitato Etico Indipendente Azienda Ospedaliero-Universitaria “Policlinico Consorziale”), Bari (Italy), approved on May 5, 2014; Regional Ethics Committee (Comitato Etico Regionale), Genoa (Italy), approved on December 9, 2014; Intercompany Ethics Committee AOU “Maggiore della Carità” (Comitato Etico Interaziendale AOU “Maggiore della Carità”), Novara (Italy), approved on July 25, 2014; Bioethical Committee of the Medical University of Warsaw (Komisja Bioetyczna przy Warszawskim Uniwersytecie Medycznym), Warsaw (Poland), approved on March 18, 2014; Bioethical Committee of the Institute of Psychiatry and Neurology (Instytut Psychiatrii i Neurologii Komisja Bioetyczna), Warsaw (Poland), approved on September 19, 2013; Ethics Committee of the Western Lisbon Hospital (Comissão de Ética do Centro Hospitalar de Lisboa Ocidental), Lisbon (Portugal), approved on April 30, 2014; Ethics Committee of the “Iuliu Hatieganu” University of Medicine and Pharmacy (Univeritatea se Medicină şi Farmacie “Iuliu Hatieganu” Comisia de Etică), Cluj-Napoca (Romania), approved on April 111, 2014; Research Ethics Committee of Principado de Asturias (Comité de Ética de la Investigación del Principado de Asturias), Oviedo (Spain), approved on June 3, 2014; Clinical Research Ethics Committee (Comité Ético de Investigación Clínica), Madrid (Spain), approved on March 3, 2015; Regional Ethics Committee of Lund (Regionala Etikprövningsnämnden Lund), Lund (Sweden), approved on August 25, 2014. The patients/participants provided their written informed consent to participate in this study.

## Author Contributions

VC and GH conceived and designed the analysis, contributed to writing, and reviewed the first draft. NP contributed to the statistical analysis and wrote the first draft. MI organized the database, performed the data analysis, and contributed to drafting and reviewing the manuscript. MS was the project coordinator, conceptualized the manuscript and the methodology, reviewed, and edited the first draft. HG contributed to formal analysis. PZ and CG collected the data and contributed to organizing the database and drafting the manuscript. MA, EB-G, AB, DC, PC, GD, JE, RG, CJ, MH, MC, PMR, PS, and MW collected the data. All authors contributed to manuscript revision and read and approved the submitted version.

## Funding

The EUDOR-A study was promoted and funded by EMOTRA AB, Sweden.

## Conflict of Interest

For the EUDOR-A study, the participating centres (MS, MI, PZ, CG, MA, EB-G, AB, DC, PC, GD, JE, RG, CJ, MH, MC, PMR, PS, and MW) received funding by EMOTRA AB, Sweden to perform the study. The remaining authors declare that the research was conducted in the absence of any commercial or financial relationships that could be construed as a potential conflict of interest.

## Publisher's Note

All claims expressed in this article are solely those of the authors and do not necessarily represent those of their affiliated organizations, or those of the publisher, the editors and the reviewers. Any product that may be evaluated in this article, or claim that may be made by its manufacturer, is not guaranteed or endorsed by the publisher.
