# Supplementary material for: A Naturalistic, European Multi-Center Clinical Study of Electrodermal Reactivity and Suicide Risk Among Patients With Depression
Source: Front Psychiatry. 2022 Jan 5;12:765128. doi: 10.3389/fpsyt.2021.765128 (PMC8766803; doi:10.3389/fpsyt.2021.765128)
Supplement: Supplementary file 1 [file Data_Sheet_1.docx]

**Supplementary Table 1. Sensitivity and specificity of EDOR test and suicide attempt at baseline for bipolar and unipolar depressed patients.**

| **Bipolar depression (F31)^a^** | | **Attempted suicide at baseline** | | | | |
| --- | --- | --- | --- | --- | --- | --- |
|  | | Yes | No | Total |  | Value (95% CI) |
| EDOR Test | Hyporeactive | TP=47 | FP=43 | 90 | Sensitivity (%) | 35.61 (24.47-44.41) |
|  | Reactive | FN=85 | TN=119 | 204 | Specificity (%) | 73.46 (65.96-80.08) |
|  | Total | 132 | 162 | 294 | PPV (%) | 52.22 (43.66-60.66) |
|  |  |  |  |  | NPV (%) | 58.33 (54.47-62.21) |
| **Unipolar depression (F32, F33)^b^** | | **Attempted suicide at baseline** | | | | |
|  | | Yes | No | Total |  | Value (95% CI) |
| EDOR Test | Hyporeactive | TP=93 | FP=116 | 209 | Sensitivity (%) | 27.93 (23.17-33.08) |
|  | Reactive | FN=240 | TN=526 | 766 | Specificity (%) | 81.73 (78.73-84.83) |
|  | Total | 642 | 333 | 975 | PPV (%) | 44.50 (38.71-50.44) |
|  |  |  |  |  | NPV (%) | 68.67 (67.01-70.28) |

TP= true positive, FP= false positive, FN= false negative, TN= true negative.

^a^ Pearson Chi-Square, non-significant

^b^ Pearson Chi-Square, p<.001

**Supplementary Table 2. Sensitivity and specificity of EDOR test and suicide attempt during follow-up for bipolar and unipolar depressed patients.**

| **Bipolar depression (F31)** | | **Attempted suicide during follow-up** | | | | |
| --- | --- | --- | --- | --- | --- | --- |
|  | | Yes | No | Total |  | Value (95% CI) |
| EDOR Test | Hyporeactive | TP=13 | FP=77 | 90 | Sensitivity (%) | 48.15 (28.67-68.05) |
|  | Reactive | FN=14 | TN=190 | 204 | Specificity (%) | 71.16 (65.33-76.52) |
|  | Total | 27 | 267 | 294 | PPV (%) | 14.44 (9.86-20.68) |
|  |  |  |  |  | NPV (%) | 93.14 (90.35-95.16) |
| **Unipolar depression (F32, F33)** | | **Attempted suicide during follow-up** | | | | |
|  | | Yes | No | Total |  | Value (95% CI) |
| EDOR Test | Hyporeactive | TP=13 | FP=196 | 209 | Sensitivity (%) | 25.53 (14.95-41.08) |
|  | Reactive | FN=36 | TN=730 | 766 | Specificity (%) | 78.83 (76.06-81.42) |
|  | Total | 49 | 926 | 975 | PPV (%) | 6.22 (3.93-9.70) |
|  |  |  |  |  | NPV (%) | 95.30 (94.47-96.01) |

TP= true positive, FP= false positive, FN= false negative, TN= true negative.

^a^ Pearson Chi-Square, p=.038

^b^ Pearson Chi-Square, non-significant

**Supplementary Table 3. Sensitivity and specificity of EDOR test and suicide attempt during follow-up for bipolar and unipolar depressed patients, excluding patients with previous suicide attempt.**

| **Bipolar depression (F31)^a^** | | **Attempted suicide during follow-up** | | | | |
| --- | --- | --- | --- | --- | --- | --- |
|  | | Yes | No | Total |  | Value (95% CI) |
| EDOR Test | Hyporeactive | TP=3 | FP=40 | 43 | Sensitivity (%) | 75.00 (19.41-99.37) |
|  | Reactive | FN=1 | TN=118 | 119 | Specificity (%) | 74.68 (67.16-81.26) |
|  | Total | 4 | 158 | 162 | PPV (%) | 6.98 (3.86-12.30) |
|  |  |  |  |  | NPV (%) | 99.16 (95.57-99.85) |
| **Unipolar depression (F32, F33)^a^** | | **Attempted suicide during follow-up** | | | | |
|  | | Yes | No | Total |  | Value (95% CI) |
| EDOR Test | Hyporeactive | TP=2 | FP=114 | 116 | Sensitivity (%) | 20.00 (2.52-55.61) |
|  | Reactive | FN=8 | TN=518 | 526 | Specificity (%) | 81.96 (78.74-84.88) |
|  | Total | 10 | 632 | 642 | PPV (%) | 1.72 (0.50-5.77) |
|  |  |  |  |  | NPV (%) | 98.48 (97.93-98.88) |

TP= true positive, FP= false positive, FN= false negative, TN= true negative.

^a^ Fisher’s Exact Test, non-significant

**Supplementary Table 4. Sensitivity and specificity of EDOR test and suicide for bipolar and unipolar depressed patients.**

| **Bipolar depression (F31)^a^** | | **Suicide** | | | | |
| --- | --- | --- | --- | --- | --- | --- |
|  | | Yes | No | Total |  | Value (95% CI) |
| EDOR Test | Hyporeactive | TP=0 | FP=90 | 90 | Sensitivity (%) | 0 (0-97.50) |
|  | Reactive | FN=1 | TN=203 | 204 | Specificity (%) | 69.28 (63.65-74.52) |
|  | Total | 1 | 293 | 294 | PPV (%) | 0 |
|  |  |  |  |  | NPV (%) | 99.51 (99.47-99.55) |
| **Unipolar depression (F32, F33)^a^** | | **Suicide** | | | | |
|  | | Yes | No | Total |  | Value (95% CI) |
| EDOR Test | Hyporeactive | TP=2 | FP=207 | 209 | Sensitivity (%) | 33.33 (4.33-77.72) |
|  | Reactive | FN=4 | TN=762 | 766 | Specificity (%) | 78.64 (75.92-81.18) |
|  | Total | 6 | 969 | 975 | PPV (%) | 0.96 (0.31-2.93) |
|  |  |  |  |  | NPV (%) | 99.48 (99.08-99.70) |

TP= true positive, FP= false positive, FN= false negative, TN= true negative.

^a^ Fisher’s Exact Test, non-significant
